# GCS as a predictor of mortality in patients with traumatic inferior vena cava injuries: a retrospective review of 16 cases

**DOI:** 10.1186/1749-7922-8-59

**Published:** 2013-12-29

**Authors:** Michael Cudworth, Angelo Fulle, Juan P Ramos, Ivette Arriagada

**Affiliations:** 1 Adult Emergency Services, Surgery, Hospital Dr. Sotero del Rio, Concha y Toro, 3459 Puente Alto, Santiago, Chile; 2 Vascular Surgery, Hospital Dr. Sotero del Rio, Concha y Toro, 3459 Puente Alto, Santiago, Chile

**Keywords:** Vascular, Trauma, Inferior vena cava, Glasgow, Injury

## Abstract

**Introduction:**

Recent research has determined Glasgow Coma Scale (GCS) to be an independent predictor of mortality in patients with traumatic inferior vena cava (IVC) injuries. The aim of this study was to evaluate the use of GCS, as well as other factors previously described as determinants of mortality, in a cohort of patients presenting with traumatic IVC lesions.

**Methods:**

A 7-year retrospective review was undertaken of all trauma patients presenting to a tertiary care trauma center with trauma related IVC lesions. Factors described in the literature as associated with mortality were assessed with univariate analysis. ANOVA analysis of variance was used to compare means for continuous variables; dichotomous variables were assessed with Fischer’s exact test. Logistic regression was performed on significant variables to assess determinants of mortality.

**Results:**

Sixteen patients with traumatic IVC injuries were identified, from January 2005 to December 2011. Six patients died (mortality, 37.5%); the mechanism of injury was blunt in one case (6.2%) and penetrating in the 15 others (93.7%). Seven patients underwent thoracotomy in the operating room (OR) to obtain vascular control (43.7%). Upon univariate analysis, non-survivors were significantly more likely than survivors to have lower mean arterial pressures (MAP) in the emergency room (ER) (45.6 +/- 8.6 vs. 76.5 +/- 25.4, p = 0.013), a lower GCS (8.1 +/- 4.1 vs. 14 +/- 2.8, p = 0.004), more severe injuries (ISS 60.3 +/- 3.5 vs 28.7 +/- 22.9, p = 0.0006), have undergone thoracotomy (83.3% vs. 16.6%, p = 0.024), and have a shorter operative time (105 +/- 59.8 min vs 189 +/- 65.3 min, p = 0.022). Logistic regression analysis revealed GCS as a significant inverse determinant of mortality (OR = 0.6, 0.46-0.95, p = 0.026). Other determinants of mortality by logistic regression were thoracotomy (OR = 20, 1.4-282.4, p = 0.027), and caval ligation as operative management (OR = 45, 2.28-885.6, p = 0.012).

**Conclusions:**

GCS, the need to undergo thoracotomy, and caval ligation as operative management are significant predictors of mortality in patients with traumatic IVC injuries.

## Introduction

Traumatic inferior vena cava (IVC) lesions represent 30% to 40% of trauma related abdominal vascular injuries [1-4]. In spite of significant advances in pre-hospital care, surgical technique, and surgical critical care, traumatic IVC lesions continue to carry a high overall mortality of 43% [1,5-11]. Roughly 30% to 50% of patients sustaining traumatic IVC injuries will die of their injuries before reaching a hospital [1,5-7,9,11,12]. Of those patients that survive long enough to be hospitalized, another 30% to 50% will decease in spite of surgical therapy and resuscitation efforts [13-15]. Penetrating trauma is the cause of 86% of IVC injuries, with blunt trauma causing only 14% of IVC injuries [1,5,7-10,14,16-18]. The IVC is anatomically divided into five segments: infra-renal (IRIVC), para-renal (PRIVC), supra-renal (SRIVC), retro-hepatic (RHIVC), and supra-hepatic (SHIVC). Overall, the most frequently injured segment is the IRIVC (39%), followed by the RHIVC (19%), SRIVC (18%), PRIVC (17%), and the SHIVC (7%) [1,5,7-10,14,16-18]. Numerous studies have analyzed factors associated with mortality in IVC lesions. Factors predictive of mortality reported include level of the IVC injury, hemodynamic status on arrival, number of associated injuries, blood loss and transfusional requirements, among others [1,5,7-10,14,16-18]. Recent work by Huerta el al described Glasgow Coma Scale (GCS) as an independent predictor of mortality in IVC trauma [5]. The aim of this study was to assess GCS, as well as other factors previously described as determinants of mortality, in a cohort of patients presenting with traumatic IVC lesions at an urban tertiary care trauma center.

## Methods

Approval for this study was obtained from the Hospital’s ethics committee. A retrospective chart review was performed from January 2005 to December 2011, of all abdominal vascular trauma patients presenting to the tertiary care trauma center at Hospital Dr. Sotero del Rio. Patients that died before operative intervention or pronounced dead on arrival were excluded. All patient charts were individually reviewed for the following parameters: demographic data, Injury Severity Score (ISS), initial systolic blood pressure in the ED (SBP), initial diastolic blood pressure in the ED (DBP), initial heart rate in the ED, admission base deficit expressed as base excess (BE), time in the ED prior to operative intervention, and GCS as determined by a chief resident or the most senior attending trauma surgeon in the trauma bay. Operative records were reviewed for mechanism and location of IVC injury, the number of associated injuries encountered, the method of vascular control and repair, the need for thoracotomy for vascular control, transfusional requirements, and operative time. Other data assessed included length of hospital stay. Statistical analysis was performed with STATA 12.1 (Stata Corp LP, College Station, TX). Data is represented as means +/- SE for univariate and logistic regression analysis, and means +/- SD for oneway ANOVA analysis of variance. P values of less than 0.05 were considered significant. Univariate analysis was performed using either Student’s T-test or one-way ANOVA analysis of variance for continuous variables and Fischer’s exact test for dichotomous variables. Outcome association with mechanism of injury, and level of injury were assessed using Kruskal–Wallis rank test. Variables achieving statistical significance on univariate analysis were included in a logistic regression model to assess variables predictive of survival. A receiver operating characteristic curve was determined to assess model fit of the regression model.

## Results

During the 7-year period from January 2005 to December 2011, sixteen traumatic IVC injuries were identified at the Hospital Dr. Sotero del Rio, Santiago, Chile (mean age = 25.6 +/- 1.9 years; ISS = 40.5 +/- 5.19; 87% male and 12% female). The mortality rate was 37.5% (6 patients). The mechanism of IVC injury was 56.2% gun shot wound (GSW) (9 patients), 37.5% stab wound (SW) (6 patients), and 6.3% blunt injury (1 patient). In our series, the initial GCS was 11.8 +/- 1.1. The number of associated injuries was 2.3 +/- 0.3, including one or more of the following: superior mesenteric vasculature, gastric, duodenum, small bowel, large bowel, splenic, pancreatic, liver, lung, diaphragm, and cardiac. Univariate analysis did not show a significant increase in mortality with any associated injury (Table 1). Non-survivors were significantly more likely to be hypotensive in the ER (ER MAP, 45.6 +/- 8.6 mmHg vs. 76.5 +/- 25.4 mmHg, p = 0.013), have a lower GCS (8.1 +/- 4.1 vs. 14 +/- 2.8, p = 0.004), have undergone thoracotomy in the OR (83.3% vs. 16.6%, p = 0.024), have a shorter operative time (105 +/- 59.8 min vs 189 +/- 65.3 min, p = 0.022), and have more severe injuries (ISS 60.3 +/- 3.5 vs 28.7 +/- 22.9, p = 0.0006) (Table 2).

**Table 1:** Distribution of associated injuries between groups

	**Survivors (n = 10)**	**Non-survivors (n = 6)**	P value*
Gastric	1 (10%)	1 (16%)	NS
Duodenum	2 (20%)	1 (16%)	NS
Small bowel	4 (40%)	2 (33%)	NS
Large bowel	1 (10%)	1 (16%)	NS
Spleen	1 (10%)	1 (16%)	NS
Kidney	3 (30%)	2 (33%)	NS
Liver	2 (20%)	1 (16%)	NS
Pancreas	1 (10%)	1 (16%)	NS
Lung	1 (10%)	1 (16%)	NS
Diaphragm	0 (0%)	1 (16%)	NS
Cardiac	2 (20%)	0 (0%)	NS
Aorta	0 (0%)	3 (50%)	NS
Superior mesenteric artery	0 (0%)	1 (16%)	NS
Splenic or iliac vein	1 (10%)	1 (16%)	NS

*NS, not significant.

**Table 2:** Significant differences between groups

	**Survivors (n = 10)**	**Nonsurvivors (n = 6)**	**P value**
ER MAP (mmHg)	76.5 +/- 25.4	45.6 +/- 8.6	0.013*
GCS	14 +/- 2.8	8.17 +/- 4.1	0.004*
Operative time (min)	189 +/- 65.3	105 +/- 59.8	0.022*
ISS	28.7 +/- 3.5	60.3 +/- 22.9	0.0006*
OR thoracotomy	20%	83.3%	0.024 +

*Oneway ANOVA analysis of variance.

+ Fischer’s exact test.

Six patients (37.5%) were managed with IVC ligation due to difficulty in obtaining adequate exposure and intraoperative hemodynamic instability, and ten patients (62.5%) were managed with simple primary repair.

Caval ligation was significantly associated with increased mortality, with five out of the six patients managed with IVC ligation deceasing (mortality: 83.3%) as opposed to one patient out of ten managed with primary repair (mortality: 16.67%, p = 0.008) (Table 3). Upon logistic regression analysis, significantly increased odds of mortality were seen with the need to undergo thoracotomy for vascular control (OR = 20, 1.4-282.4, p = 0.027), and the use of caval ligation as operative management (OR = 45, 2.28-885.6, p = 0.012) (Table 4). GCS as a linear scale displayed an inverse relation with the risk of mortality expressed as a binary outcome. Upon linear regression analysis, GCS was a significant inverse predictor of mortality, (p = 0.005) (Table 5). Upon logistic regression, a higher GCS was associated with significantly lower odds of mortality (OR = 0.6, 0.46-0.95, p = 0.026). ROC curves after logistic regression as a measure of model fit were 0.85 for GCS, 0.86 for caval ligation as operative management, and 0.81 for thoracotomy. In our cohort of patients, neither the mechanism of injury, nor the level of the IVC injury were significantly associated with an increase in mortality (Tables 6 and 7). No statistically significant differences existed among non-survivors and survivors for BE on admission (-19.4 +/- 8.3 vs. -12.7 +/- 6.1, p = 0.08), total number of associated injuries (2.8 +/- 1.4 vs. 1.9 +/- 0.9, p = 0.15), transfusional requirements expressed as packed red blood cells (PRBC) (7.09 +/- 2.5 vs. 7.23 +/- 2.7, p = 0.9), or time to surgical treatment (19.5 +/- 6.9 min vs. 32.3 +/- 18.5 min, p = 0.13). Non-survivors mainly died on the operating table due to massive hemorrhage that was impossible to control operatively, with subsequent cardiac arrest. The mean hospital stay of survivors was 24.5 +/- 14.2 days.

**Table 3:** **Mortality by operative management** (**caval ligation versus simple repair**)

**Operative management**	**Number of patients**	**Number of deaths**	**ISS +**	Mortality rate*
IVC ligation	6 (37.5%)	5	59 +/- 10.1	83.3%
Simple repair	10 (62.5%)	1	29.5 +/- 1.2	16.6%

+P value = 0.002, Student’s T-test.

*P value = 0.008, Fischer’s exact test.

**Table 4:** Significant predictors of mortality by logistic regression

	**OR**	**P value**	**Confidence interval**	Area under ROC curve*
Thoracotomy	20	0.027	1.4-282.4	0.81
IVC ligation	45	0.012	2.28-885.6	0.86
**Significant inverse predictors of mortality by logistic regression**				
	**OR**	**P value**	**Confidence interval**	Area under ROC curve*
GCS	0.6	0.026	0.46-0.95	0.85

*Area under ROC curve as a measure of model fit.

**Table 5:** GCS as a determinant of mortality by linear regression

	**Beta coefficient**	P value*	**R**^**2**^** +**
GCS	-0.07	0.005	0.44
Intercept	1.27		

*Inverse relation between GCS and mortality by linear regression.

+ R-squared as a measure of model fit.

**Table 6:** Mortality by mechanism of injury

**Mechanism**	**Number**	Mortality rate*
Blunt	1 (6.25%)	0%
GSW	9 (56.25%)	44.4%
SW	6 (37.5%)	33.3%
Total	16	37.5%

*P = 0.6 (NS), Kruskal–Wallis analysis of variance rank test.

**Table 7:** Mortality by number of injuries and IVC level of injury

**Level of injury**	**Number of injuries**	**Number of deaths**	**Mortality rate**
Infrarenal	4 (25%)	1	25%
Pararenal	4 (25%)	1	25%
Suprarenal	5 (31.2%)	3	60%
Retrohepatic	1 (6.25%)	1	100%
Intrapericardial	2 (12.5%)	0	0%
	P value = 0.8 (NS)*		P value = 0.3 (NS)*

*Kruskal–Wallis analysis of variance rank test.

## Discussion

Traumatic IVC injuries are a relatively rare event, occurring in only up to 5% of penetrating injuries and only up to 1% of blunt abdominal trauma [8]. Nonetheless, IVC trauma continues to present a formidable challenge to trauma surgeons, carrying an overall high mortality rate in spite of recent improvements in pre-hospital care, resuscitation upon arrival at a trauma center, diagnostic imaging, and timely surgical care. Our overall mortality rate for IVC trauma (37.5%) is consistent with previous reports of IVC trauma mortality ranging from 21% to 56%, with an overall mortality rate of 43% [1,5,7-10,14,16-18]. Previous reports have described predictors of mortality to be level of injury, shock on admission, timing of diagnosis to definitive management, blood loss, requirements for blood transfusions, associated injuries, ED thoracotomy, preoperative lactate and base deficits, ISS, and GCS [1,5,7-10,16-18]. In our cohort, we found statistically significant associations with the risk of mortality with hypotension upon arrival at the ER, thoracotomy, operative time, injury severity expressed as ISS, and GCS. There was a trend towards ascending mortality as the level of injury approached the heart, however we were unable to find a statistically significant relation between level of injury and mortality. This is likely due to the small size of our cohort, and the fact that the two patients in our series with intra-perdicardial lesions, both survived. Upon regression analysis, significant predictors of mortality were thoracotomy, IVC ligation as operative management, and GCS.

GCS as an independent predictor of mortality in IVC trauma has been previously described in a patient population with a higher incidence of blunt trauma (28%) than our patient cohort (6.3%) [5]. Nonetheless, in our patient cohort presenting with a high incidence of penetrating IVC trauma (93.7%), logistic regression confirmed GCS is significantly associated with mortality. In our cohort, patients did not sustain major head injuries, thus the significant association GCS demonstrated with mortality likely reflects substantial hemodynamic compromise, as has been previously proposed [5].

The other determinants of mortality in our regression model were thoracotomy and to have undergone IVC ligation instead of simple suture repair. The use of thoracotomy to obtain vascular control likely suggests more extensive vascular injuries, which is consistent with the fact non-survivors had significantly more severe injuries as expressed by a higher ISS. Significantly better survival has been previously described in IVC injuries treated with IVC ligation [1], and thus our results must be interpreted with caution. However, in our cohort IVC ligation was utilized as a salvage method to treat vascular injuries not amenable to primary repair or when the surgical team faced difficulty in obtaining adequate exposure in a patient at risk of exsanguination. Patients treated with IVC ligation had more severe injuries as reflected by a significantly higher ISS (Table 3).

Our study has several limitations, including our small sample size and its retrospective nature. However our results are relevant as we confirm GCS as a predictor of mortality in patients with traumatic IVC injuries. This study, along with others, point to the relevance of GCS as a predictor of mortality in patients with IVC trauma, of both blunt and penetrating etiology. Further prospective studies are needed to confirm the validity of GCS along with other previously described determinants of mortality in IVC trauma. Likewise, management protocols need be established to decrease the high mortality rate that is still seen with traumatic IVC injuries, which has not improved in spite of improved resuscitation and pre-hospital care.

## Conclusions

In spite of being a relatively rare event, trauma related IVC injuries present a formidable challenge to the trauma surgeon, with a high overall mortality rate of 43%, which has not changed in recent years despite vast improvements in pre-hospital transport time and care, hospital resuscitation and surgical critical care. Our results confirm GCS is an independent predictor of mortality in IVC trauma. Other significant determinants of mortality in our cohort were the use of thoracotomy, and the use of IVC ligation as operative management. Further prospective studies are needed to confirm the validity of the described determinants of mortality in IVC trauma. Management protocols need be established to decrease the high mortality rate still carried by traumatic IVC injuries.

## Abbreviations

IVC: Inferior vena cava; GCS: Glasgow coma scale; ISS: Injury severity score; ED: Emergency department; OR: Operating room; BE: Base excess; MAP: Mean arterial pressure; SBP: Systolic blood pressure; DBP: Diastolic blood pressure; IRIVC: Infra-renal vena cava; PRIVC: Para-renal vena cava; SRIVC: Supra-renal vena cava; RHIVC: Retro-hepatic vena cava; SHIVC: Supra-hepatic vena cava; ROC: Receiver operator curve; NS: Not significant.

## Competing interests

The author’s declare that they have no competing interests.

## Authors’ contributions

All authors: 1) have made substantial contributions to conception and design, or acquisition of data, or analysis and interpretation of data; 2) have been involved in drafting the manuscript or revising it critically for important intellectual content; 3) have given final approval of the version to be published. MC: Study conception and design, acquisition of data, analysis and interpretation of data, drafting of manuscript. JR: Study conception and design, acquisition of data, analysis and interpretation of data, drafting of manuscript. AF: Study conception and design, acquisition of data, critical revision of manuscript. IA: Study conception and design, acquisition of data, analysis and interpretation of data, critical revision of manuscript. All authors have read and approved the final manuscript.
